# Growth, Biochemical Traits, Antioxidant Enzymes, and Essential Oils of Four Aromatic and Medicinal Plants Cultivated in Phosphate-Mine Residues

**DOI:** 10.3390/plants13182656

**Published:** 2024-09-22

**Authors:** Khadija Ait Elallem, Widad Ben Bakrim, Abdelaziz Yasri, Ali Boularbah

**Affiliations:** 1 Laboratoire Bioressources et Sécurité Sanitaire des Aliments, Faculté des Sciences et Techniques, Université Cadi Ayyad, Marrakech 40000, Morocco; khadija.aitelallem@um6p.ma; 2 Biomass Valorization and Biorefinery Laboratory, Biodiversity & Plant Sciences Division, Mohammed VI Polytechnic University, Benguerir 43150, Morocco; widad.benbakrim@um6p.ma (W.B.B.); abdelaziz.yasri@inra.ma (A.Y.); 3 African Sustainable Agriculture Research Institute, Mohammed VI Polytechnic University, Laâyoune 70000, Morocco; 4 Institut National de la Recherche Agronomique (INRA), Rabat 10090, Morocco; 5 Center of Excellence for Soil and Fertilizer Research in Africa, College of Agriculture and Environmental Sciences, Mohammed VI Polytechnic University, Benguerir 43150, Morocco

**Keywords:** phosphate-mine residues, revegetation, aromatic and medicinal plants, essential oil

## Abstract

Revegetation emerges as a promising approach to alleviate the adverse impacts of mining residues. However, it is essential to evaluate the characteristics of these materials and select suitable plant species to ensure successful ecosystem restoration. This study aimed to investigate the effects of phosphate-mine residues (MR) on the growth, biochemical properties, and essential oil concentration of *Rosmarinus officinalis* L., *Salvia Officinalis* L., *Lavandula dentata* L., and *Origanum majorana* L. The results showed that *R. officinalis* L. appeared to be particularly well-suited to thriving in MR soil. Our finding also revealed that *L. dentata* L., *O. majorana* L., and *S. officinalis* L. grown in MR exhibited significantly lower growth performance (lower shoot length, smaller leaves, and altered root structure) and higher antioxidant activities, with an alterations of photosynthetic pigment composition. They showed a decrease in total chlorophylls when grown on MR (0.295, 0.453, and 0.562 mg g^−1^ FW, respectively) compared to the control (0.465, 0.807, and 0.808 mg g^−1^ FW, respectively); however, they produced higher essential oil content (1.8%, 3.06%, and 2.88%, respectively). The outcomes of this study could offer valuable insights for the advancement of revegetation technologies and the utilization of plant products derived from phosphate-mine residues.

## 1. Introduction

Phosphate mining is a widespread industry that plays a significant role in meeting the global demand for agricultural fertilizers. However, it often results in the degradation of natural ecosystems. Phosphate-mining activities are also known to generate vast quantities of waste rocks [1,2], causing long-term environmental consequences. In addition, these activities often create visual disturbances for locals living around them [3]. In the process of extraction, the removal of overburden and intercalation layers is conducted to access the phosphate ore, and these materials are subsequently deposited in piles [2]. These spoil piles are then reused for reinstating the excavated site, where they are leveled to establish continuous horizontal surfaces, shaping the post-mining terrain. Revegetating these lands represents a crucial step and a difficult challenge for ecological restoration and advancing sustainable rehabilitation objectives [4]. The reestablishment of vegetation serves as a pivotal strategy in enhancing soil stability, mitigating erosion, and promoting the recovery of biodiversity. Furthermore, the revegetation process holds the potential to facilitate carbon sequestration, improve water quality, and secure a habitat for wildlife [5].

A crucial step in the revegetation of phosphate mines is selecting the proper plant species. Native plants that have adapted to those environments are frequently preferred due to their higher chances of thriving and positively impacting the environment [6]. However, due to the physicochemical properties of the substrate, such as alkalinity, saline–sodic conditions, low organic matter content, low nutrient availability, negligible soil microbial activity, poor structure, and low water retention capacity [1,7,8], natural regeneration and plant growth in such degraded areas is slow and frequently impossible. For the revegetation process, it is frequently required to artificially introduce suitable plant species, which may then be permitted to thrive and spread on their own. In order to properly revegetate mined sites, it is crucial to first identify and choose the right species [6].

*R. officinalis* L. (rosemary), *S. officinalis* L. (sage), *L. dentata* L. (French lavender), and *O. majorana* L. (sweet marjoram) are commonly cultivated medicinal and aromatic plants that have significant economic and ecological importance. They are valued for their essential oil content, which contains bioactive compounds with potential uses in the pharmaceutical, food, and cosmetic industries. Those species can show resistance strategies [9] being able to acclimate to adverse environmental conditions and to overcome the negative effects caused by the low quality of the substrate. Several studies have investigated the effects of various stress factors on the growth, morphology, and biochemical characteristics of these plants, including heavy metal toxicity, drought, and salinity stress [9,10,11,12,13]. However, there is limited research on the effects of phosphate-mine residues on these plants.

The growth of aromatic plants in environments impacted by mining activities relies on a combination of adaptive mechanisms that enable these plants to persist and adapt to challenging environmental conditions. These mechanisms encompass physiological, biochemical, and molecular strategies that collectively aim to mitigate the adverse effects of stressors associated with mining degradation [9,14,15]. Metabolic adjustments involve alterations in various metabolic pathways to maintain energy production, synthesize protective compounds, and redirect resources for stress tolerance [16]. The specific nature of these adjustments can vary depending on the type of stress encountered, such as drought, salinity, temperature extremes, nutrient deficiency, or exposure to pollutants. When faced with inadequate nutrient availability for example, plants activate intricate processes aimed at ensuring their survival and continued growth. These mechanisms encompass alterations in root architecture to enhance nutrient uptake, the secretion of organic acids to facilitate the solubilization of nutrients from the surrounding soil, and the formation of symbiotic relationships with mycorrhizal fungi for improved nutrient absorption [17,18,19] Additionally, plants exhibit altered nutrient transport within their tissues, prioritizing essential nutrients and redistributing them from older to younger tissues [20].

Under stress, plants can modulate photosynthesis to optimize energy production and minimize ROS generation. In some cases, they may downregulate photosynthesis and reduce their chlorophyll content to reduce water loss and metabolic demands [21]. This adjustment helps to optimize light absorption while preventing the wasteful accumulation of chlorophyll in conditions where it cannot be effectively used. Stressed plants often exhibit stomatal closure to regulate gas exchange [22]. This helps conserve water and control transpiration, but it can also limit CO_2_ influx for photosynthesis. Plants can alter their leaf structure under nutrient stress. For instance, leaves might become smaller, and their surface area might be reduced to minimize water loss through transpiration [23]. These adjustments in the photosynthetic process help plants maintain their energy production and overall growth despite limited nutrient availability.

To cope with nutrient deficiency and other stressors, aromatic plants may accumulate compatible solutes, such as proline and sugars. These compounds acts as osmo-protectants and antioxidants, help maintain cellular osmotic balance, minimize damage caused by stress-induced water loss, stabilize the subcellular structures, and contribute to in the elimination of free radicals [24]. Aromatic plants are known for producing secondary metabolites, such as phenolic compounds, like flavonoids, phenols, terpenoids, and alkaloids, and antioxidants, which serve as defense mechanisms against oxidative stress [25,26]. Stress has an impact on the protein profile of plants, mainly influencing the proteins involved in defense against oxidative stress. Amino acids play roles beyond protein synthesis during stress. For example, proline acts as an osmolyte and an ROS scavenger. Some amino acids serve as precursors for the synthesis of secondary metabolites with protective functions [27,28]. Stress can alter lipid metabolism, leading to changes in membrane composition and fluidity. Unsaturated fatty acids and membrane lipids, which enhance membrane stability under stress, might be produced in greater quantities [29].

Under stress, plants increase the production of reactive oxygen species (ROS). ROS, including hydrogen peroxide (H_2_O_2_), superoxide radicals (O_2_^·−^), and hydroxyl radicals (OH·), are generated as natural byproducts of various metabolic pathways. However, excessive ROS accumulation can lead to oxidative stress, damaging cellular components and impairing plant growth and development [14,30,31,32]. To regulate ROS production, maintain redox homeostasis, and counteract oxidative damage caused by excessive ROS, plants increase the activity of antioxidant enzymes, such as superoxide dismutase (SOD), catalase (CAT), and peroxidases (PODs). These enzymes scavenge ROS and convert them into less harmful molecules, preventing ROS-induced damage. Non-enzymatic antioxidants, like ascorbate, glutathione, and phenolic compounds, are also synthesized or accumulated [33,34,35,36]. These antioxidants directly neutralize ROS and contribute to redox balance. These coordinated strategies enable plants to manage ROS levels effectively and mitigate the detrimental effects of oxidative stress, thereby promoting their growth and survival in environments affected by mining activities. Once produced in excess without effective scavenging mechanisms, ROS can lead to oxidative damage in the cell membrane, which can be assessed through the concentration of malonaldehyde (MDA) in plant tissues [23]. The accumulation of MDA suggests an imbalance between ROS production and the antioxidant defense system.

Environmental stresses exert comparable effects on both the content and composition of essential oils (EO) in numerous aromatic plants, consequently influencing their overall quality [9]. Gaining a deeper comprehension of how stress impacts the production and constitution of EO holds significant importance. This comprehension becomes essential when considering the utilization of degraded land within challenging environmental contexts, with the aim of augmenting production and enhancing the quality of EO.

This work aimed to analyze specific parameters related to the mechanisms by which four aromatic plants overcome the extreme stress caused by nutritional constraints in the phosphate-mine residue and its poor structure. Our findings contribute important information to the overall understanding of these mechanisms. We investigated the effects of phosphate-mine residues on the growth, morphology, and biochemical characteristics of *R. officinalis* L., *S. officinalis* L., *L. dentata* L., and *O. majorana* L., with a particular focus on the content and chemical composition of their essential oil. This study can provide valuable insights into the potential use of these important medicinal and aromatic plants in revegetation of the phosphate-mine residue and ensure sustainable land use practices in phosphate mining regions. We hypothesize that these species will provide efficient photosynthetic, morphological, and biochemical responses to the different stresses due to the low quality of the substrate, coupled with reactive oxygen species (ROS) scavenging mechanisms when grown in the phosphate-mine residue. In addition, it is expected that the EO yield of those species will be enhanced; however, the EO composition will be substantially affected when exposed to the nutritional limitations of the phosphate-mine residue.

## 2. Results

### 2.1. Physico-Chemical Properties of the Mine Residues

The main characteristics of the used residue and agriculture soil are shown in Table 1 and Table 2.

Soil texture is one of the most common features used by scientists to describe soil. The texture of the control soil was loam, whereas the studied soil (MR) was classified as sandy clay loam. The results show that the studied materials have alkaline pH values (8.92 for MR and 8.27 for C agricultural soil). All samples have low electrical conductivity (EC), indicating no salinity in the mining residue (0.56 mS cm^−1^) and agricultural soil. The residue showed a very low total organic matter content (which did not exceed 0.51%) compared to the control soil (2.01%). The assimilable phosphorus in the mining residue (18.92 mg P_2_O_5_ kg^−1^) was significantly lower than that observed in the control soil (45.67 mg P_2_O_5_ kg^−1^). The total Kjeldahl nitrogen content was low in all study samples, with the highest value observed in the control soil (0.14%). Exchangeable K^+^ was significantly higher in C (162.28 mg K kg^−1^) compared to the values detected in mine residue (26.15 mg K kg^−1^).

These results showed that phosphate-mine residue represents a low fertility medium. Physicochemical and mineralogical analyses revealed that these soils could be considered as alkaline calcareous materials characterized by low nitrogen and potassium contents, with a moderate level of available phosphorus and a low organic matter content. The low organic matter and poor nutrient content are the major factors limiting plant development in mine residues.

### 2.2. Growth Parameters of R. officinalis L., S. officinalis L., L. dentata L., and O. majorana L. Cultivated in Phosphate-Mine Tailing

**Figure 1:**
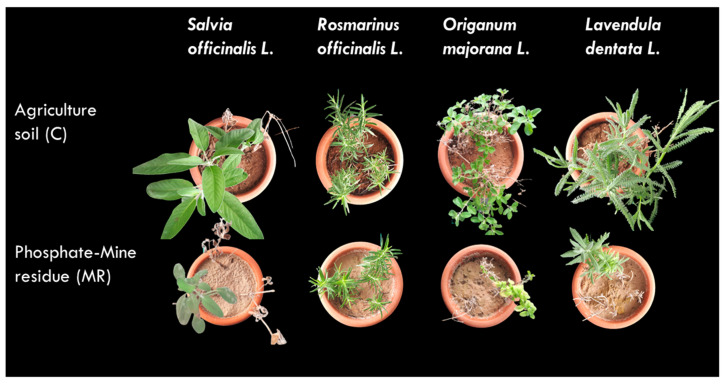
Change in the survival and the growth of the aerial parts of *R. officinalis* L., *S. officinalis* L., *L. dentata* L., and *O. majorana* L. cultivated in phosphate-mine residue and control soil.

The cultivation success of *R. officinalis* L., *S. officinalis* L., *L. dentata* L., and *O. majorana* L. was higher in the agriculture soil compared to the phosphate-mine residue (MR), where the plant growth and survival was lowest (Figure 1). The best height growth (∆SL) of the aerial part the aromatic and medicinal plants occurred in the agriculture soil (Table 3) for *S. officinalis* L. and *L. dentata* L.; however, no significant difference was observed between the ∆SL (∆SL = shoot length after harvest − initial shoot length) of *R. officinalis* L. and *O. majorana* L. grown in agriculture soil and phosphate-mine residue (*p* < 0.05). Despite the comparable value of shoot length (9 cm in C and 8.5 in MR), *O. majorana* L. grown in phosphate-mine residue showed negative phenotypic changes compared with the control plants. The leaves were characterized by a light green color, which could be due to a lower leaf photosynthetic pigment content (Figure 1).

The studied aromatic and medicinal plants showed significant differences in shoot fresh weight (SFW) among growth media (Table 3). The SFW remained low in phosphate-mine residue for *S. officinalis* L., *L. dentata* L., and *O. majorana* L., with the exception of *R. officinalis* L., where the SFW was not significantly different between the plant grown on C soil and the one grown on MR. Similarly, the four studied plants exhibit a slight decrease in shoot dry weight (SDW) when grown on MR; however, *O. majorana* L. was the only plant species where a significant difference was observed between the growth substrates, with a value of 2.68 g in C soil compared to 0.52 g in MR. From a morphological standpoint, *R. officinalis* L. tended to develop properly in both growth mediums (Figure 1, Table 3).

The results indicate that these plant species generally exhibit better growth in control soil conditions compared to phosphate-mine residues (except for *R. officinalis* L.). The findings contribute to our understanding of the potential impacts of phosphate-mine residues on plant growth, emphasizing the importance of appropriate soil management strategies for sustainable land use.

### 2.3. Root System Parameters and Architecture

**Figure 2:**
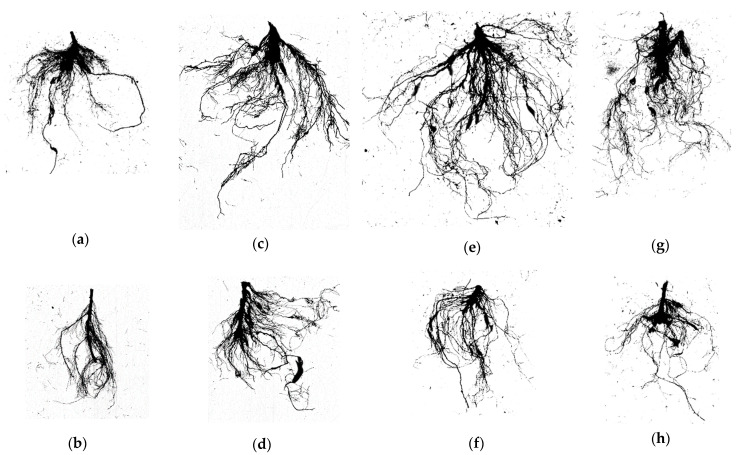
WinRHIZO image of *O. majorana* L. (**a**) C and (**b**) MR *S. officinalis* L; (**c**) C and (**d**) MR; *L. dentata* L. (**e**) C and (**f**) MR; *R. officinalis* L. (**g**) C and (**h**) MR) roots. C: Agriculture soil; MR: mine residue.

The effects of mine residue (MR) on root parameters varied among the studied plant species compared to C soil, as presented in Figure 2.

The comparison of root parameters between *L. dentata* L., *R. officinalis* L., *O. majorana* L., and *S. officinalis* L., in the C soil and MR soil types revealed notable variations in their responses to the soil change (Table 4, Figure 2).

Among the species, *L. dentata* L. appeared to be particularly sensitive to MR as a growth substrate. The *L. dentata* L. plants exhibited a slightly lower root fresh weight (RFW) compared to the control. *L. dentata* L. plants grown in C soil displayed substantially longer roots (1903.582 cm), a larger surface area (280.587 cm^2^), greater root volume (3.323 cm^3^), and higher root fitness (572.721 cm cm^−3^) compared to those in MR (595.386 cm, 119.004 cm^2^, 1.907 cm^3^, and 321.449 cm cm^−3^, respectively). The root dry weight (RDW) and root average diameter (RAD) of *L. dentata* L. were slightly higher in the MR (1.009 g and 0.635 mm) than in the C soil (0.970 g and 0.478 mm), although the difference was not statistically significant between the two soil types. This suggests that the presence of MR strongly impeded root development and growth in *L. dentata* L.

In *R. officinalis* L., RFW, RDW, and RAD were slightly higher in MR soil (2.85 g, 1.304 g, and 0.615 mm) than those in C soil (2.77 g, 1.216 g, and 0.444 mm), while there were no statistically significant differences between C soil and MR soil, suggesting comparable root biomass in both soil types. However, *R. officinalis* L. exhibited significantly longer roots (1174.388 cm), higher root volume (RV) (1.868 cm^3^), and higher root fineness (RF) (689.17 cm cm^−3^) in C soil compared to MR soil (395.0666 cm, 1.175 cm^3^, and 349.486 cm cm^−3^, respectively), indicating decreased root growth and development in the MR soil. Moreover, the RSA of *R. officinalis* L. was significantly higher in C soil (164.116 cm^2^) compared to MR soil (75.829 cm^2^).

*O. majorana* L. plants, on the other hand, displayed a significantly lower RFW, RDW, root length (RL), root surface area (RSA), and RV in MR soil (1.794 g, 0.495 g, 488.611 cm, 74.09 cm^2^, and 0.92 cm^3^) compared to the C soil (2.591 g, 0.881 g; 1132.676 cm, 172.465 cm^2^, and 2.091 cm^3^). However, there were no significant differences in RAD and RF between plants grown in the tested soil types.

*S. officinalis* L. displayed lower values in most parameters among the plant species. Likewise, *S. officinalis* L. exhibited negative responses when grown in MR soil, with significantly higher RFW, RL, RSA, and RV in C soil compared to MR soil. No significant differences were observed in RAD and RF between plants from the two soil types.

The RV and RSA were significantly impacted in all species, this suggests a smaller volume and surface area available for nutrient uptake in the MR soil (Figure 2). The differences in root average diameter between plants from MR and C were not significant for these species, indicating that the thickness of the roots was less affected by the presence of MR.

These variations in root parameters among the plant species suggest species-specific responses to soil type. *R. officinalis* L. appeared to be particularly well-suited to thriving in MR soil, with comparable root development when compared with growth in C soil. *L. dentata* L. exhibited a decrease in most parameters in MR soil. *O. majorana* L. and *S. officinalis* L. also experienced negative effects on root parameters in MR, albeit to a lesser extent. C soil generally promoted higher values of root mass, length, surface area, volume, and, in some cases, average diameter, indicating enhanced root development and nutrient uptake potential. These findings highlight the adverse impact of MR on root characteristics and plant performance across the studied species. The presence of MR restricted root growth, nutrient absorption, and water uptake, ultimately affecting the overall vigor and health of the plants. It is important to consider these negative effects when assessing the suitability of mine waste rock environments for plant growth and to develop appropriate strategies for reclamation and restoration efforts.

### 2.4. Effect of Substrate on Photosynthetic Pigments Content

Chlorophyll *a*, chlorophyll *b*, carotenoids, and total chlorophylls variation in plants grown on phosphate-mine residue (MR) and control soil (C) are presented in Table 5. Additionally, two ratios, namely the chlorophylls (*a* + *b*)/carotenoids ratio and chlorophyll *a*/*b* ratio, were calculated, and the results are provided.

The amount of chlorophyll *a* significantly reduced in plants grown on MR when compared to the plants grown on C soil (*p* ≤ 0.001). The results shows that chlorophyll a slightly decreased in *L. dentata* L. from 0.367 to 0.217 mg g^−1^ FW and decreased in *O. majorana* L. and *S. officinalis* L. from 0.650 to 0.343 mg g^−1^ FW and from 0.640 to 0.430 mg g^−1^ FW, respectively. However, no significant difference was observed on the level of chlorophyll *a* between *R. officinalis* L. grown on MR and the sample grown on C soil. Likewise, the level of chlorophyll *b* was significantly decreased (*p* ≤ 0.001) in *L. dentata* L., *O. majorana* L., and *S. officinalis* L. grown on MR when compared to the control plants. *R. officinalis* L. plants displayed a slight increase in chlorophyll *b* content in MR compared to control soil. *L. dentata* L., *O. majorana* L., and *S. officinalis* L. plants showed a notable decrease in total chlorophylls (Chl*a* + Chl*b*) when grown on MR compared to C, with values of 0.465, 0.807, and 0.808 mg g^−1^ FW in C soil compared to 0.295, 0.453, and 0.562 mg g^−1^ FW in MR, respectively. In contrast, *R. officinalis* L. plants showed comparable total chlorophylls level in both MR and C soil (0.476 and 0.465 mg g^−1^ FW, respectively).

Regarding carotenoid content, *L. dentata* L. and *R. officinalis* L. plants demonstrated higher levels in MR compared to C soil. *L. dentata* L. plants exhibited a carotenoids value of 0.034 mg g^−1^ FW in MR, whereas it was 0.026 mg g^−1^ FW in C. Similarly, *R. officinalis* L. plants displayed an increase in carotenoid content in MR compared to C soil. On the other hand, *O. majorana* L., and *S. officinalis* L. plants exhibited reduced carotenoid content in MR compared to C soil.

Two ratios were calculated to provide further insights into the pigment composition and stability under stress conditions. The chlorophyll *a/b* ratio represents the relative abundance of chlorophyll *a* to chlorophyll *b*. *L. dentata* L. and *O. majorana* L. plants displayed lower ratios in MR compared to C, indicating a relatively higher proportion of chlorophyll *b* in MR. Likewise, *R. officinalis* L. and *S. officinalis* L. plants exhibited a slight decrease in the ratio in MR compared to C soil. The chlorophylls (*a* + *b*)/carotenoids ratio indicates the relative abundance of chlorophylls to carotenoids. The ratio value increased in *O. majorana* L. and *S. officinalis* L. from 11.815 to 17.828 and 10.627 to 12.148, respectively, and decreased in *L. dentata* L. and *R. officinalis* L. from 18.520 to 8.596 and from 9.101 to 7.792, respectively, suggesting a decreased proportion of chlorophylls relative to carotenoids in MR.

The results demonstrate that the growth of plants on the phosphate-mine residues (MR) can lead to variations in chlorophyll *a*, chlorophyll *b*, carotenoid content, and the ratios of chlorophylls to carotenoids and chlorophyll *a* to chlorophyll *b*. These variations indicate alterations in pigment composition and may reflect the physiological responses of plants to the different soil types.

### 2.5. Effect of Phosphate-Mine Tailing on the Accumulation of Proline, Total Phenolic Content, Flavonoids, Total Soluble Sugars, Total Soluble Proteins Content, and Lipid Peroxidation Levels

**Figure 3:**
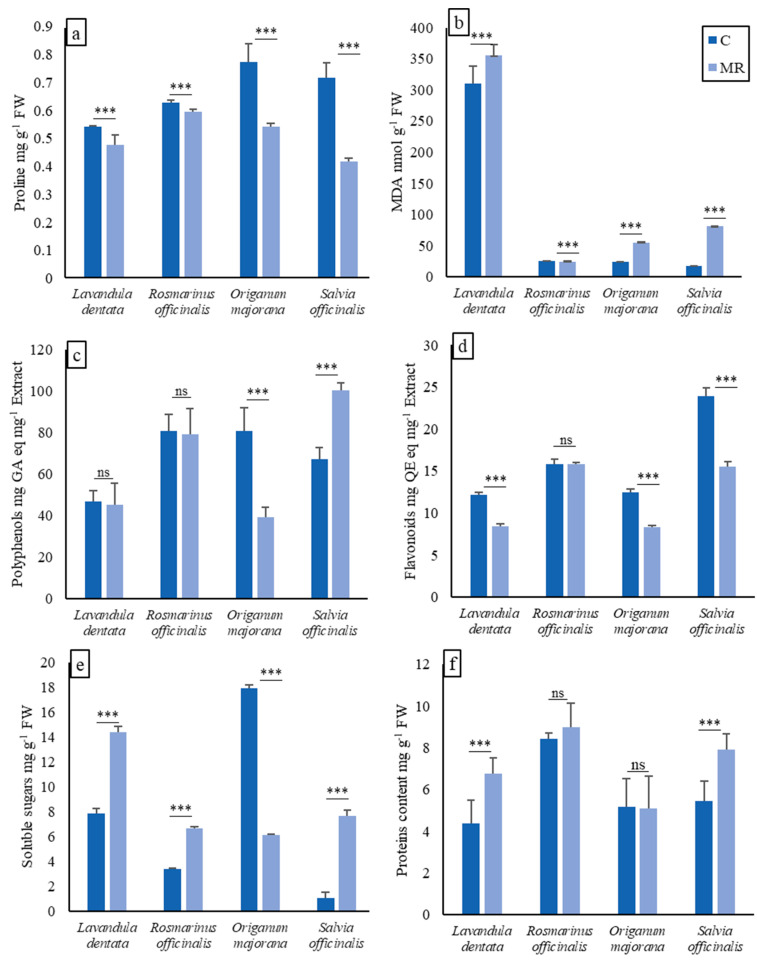
Change in (**a**) proline, (**b**) MDA, (**c**) polyphenols, (**d**) flavonoids, (**e**) soluble sugars, and (**f**) proteins content of *R. officinalis* L., *S. officinalis* L., *L. dentata* L., and *O. majorana* L. cultivated in phosphate-mine residues and control soil. C: Agriculture soil; MR: mine residue. Bars with superscript asterisks are statistically different (ns: not significant; ≤0.001: ***) using Student’s *t*-test.

The results presented in Figure 3 show the variation in proline, MDA, polyphenols, flavonoids, soluble sugars, and proteins content among the four plant species grown in mine residue soil (MR) compared to control soil (C).

The variation in proline accumulation suggests that different plant species respond differently to the soil type, particularly in relation to stress-induced proline synthesis. While *L. dentata* L. and *R. officinalis* L. exhibited a slightly lower proline content in mine residue soil (0.478 and 0.599 µg mg^−1^, respectively) compared to control soil (0.544 and 0.631 µg mg^−1^, respectively), *O. majorana* L. and *S. officinalis* L. displayed a significantly lower proline content in mine residue soil (0.544 and 0.420 µg mg^−1^, respectively) compared to control soil (0.779 and 0.721 µg mg^−1^, respectively).

The malondialdehyde (MDA) content varied among the different plant species under the two soil conditions. *L. dentata* L., *O. majorana* L., and *S. officinalis* L. showed a substantial increase in MDA content in mine residue soil (355.43, 55.16, and 80.59 nmol g^−1^ FW, respectively) compared to control soil (310.14, 23.52, and 17.50 nmol g^−1^ FW, respectively). Conversely, *R. officinalis* L. exhibited similar MDA contents in both soil types. These findings suggest that plant species respond differently to the soil conditions, particularly in relation to oxidative stress and lipid peroxidation, as indicated by MDA content. The variations observed in MDA content may be attributed to the specific adaptations and responses of these plant species to different soil types.

The total polyphenols content of *L. dentata* L. and *R. officinalis* L. did not remarkably change between C (46.706 and 80.904 µg mg^−1^ extract, respectively) and MR (45.250 and 78.977 µg mg^−1^ extract, respectively). On the other hand, *O. majorana* L. displayed a significantly higher polyphenols content in C (80.580 µg mg^−1^ extract) compared to a noticeably lower content in MR (39.147 µg mg^−1^ extract. *S. officinalis* L., however, demonstrated a reverse trend, with a higher polyphenols content in MR (100.398 µg mg^−1^ extract) compared to C (67.052 µg mg^−1^ extract). When comparing the variations in plant flavonoid content in different soil types (mine residue and control soil), we observed that *L. dentata* L., *O. majorana* L., and *S. officinalis* L. exhibited a lower flavonoids content in mine residue soil (8.39, 8.33, and 15.55 µg mg^−1^ extract, respectively) compared to control soil (12.19, 12.44, and 23.96 µg mg^−1^ extract, respectively). The highest flavonoids content was observed in *S. officinalis* L., particularly in control soil. However, *R. officinalis* L. showed a similar flavonoids content in both mine residue soil (15.83 µg mg^−1^ extract) and control soil (15.81 µg mg^−1^ extract).

Soluble sugars content was significantly increased when *L. dentata* L., *R. officinalis* L., and *S. officinalis* L. were grown in the mine residue (14.37, 6.67, and 7.64 mg g^−1^ FW, respectively). The accumulation of soluble sugars, however, significantly decreased by 66% in *O. majorana* L. when it was grown in the mine residue compared to the control soil. The maximum accumulation of sugars in the control soil (17.94 mg g^−1^ FW) was observed in *O. majorana* L., while in mine residue, the maximum accumulation of sugars was 14.37 mg g^−1^ FW, which was recorded in *L. dentata* L. Likewise, the proteins content was significantly increased in the *L. dentata* L. and *S. officinalis* L. grown in the mine residue (6.75 and 7.91 mg g^−1^ FW, respectively). However, no significant difference in the proteins level was observed in *R. officinalis* L. and *O. majorana* L. between plants from the mine residue and control soil.

### 2.6. Enzymatic Activity Responses of R. officinalis L., S. officinalis L., L. dentata L., and O. majorana L. Cultivated in Phosphate-Mine Tailing

**Figure 4:**
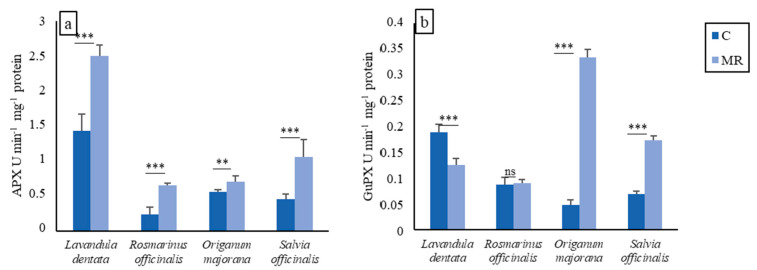
Change in enzymatic activities of (**a**) APX and (**b**) GuPX of *R. officinalis* L., *S. officinalis* L., *L. dentata* L., and *O. majorana* L. cultivated in phosphate-mine residue and control soil. C: Agriculture soil; MR: mine residue; APX: ascorbate peroxidase; GuPX: guaiacol peroxidase. Bars with superscript asterisks are statistically (ns: not significant; ≤0.01: **; ≤0.001: ***) using Student’s *t*-test.

The antioxidant enzyme activities of the four plants are given in Figure 4.

In the control group, ascorbate peroxidase (APX) activities were 0.23, 0.45, 1.42, and 0.56 U min^−1^ mg^−1^ protein in the leaves of *R. officinalis* L., *S. officinalis* L., *L. dentata* L., and *O. majorana* L., respectively. In comparison, APX activities in the leaves of all plants cultivated in phosphate-mine significantly rose compared to those grown in the control soil, with the exception of *O. majorana* L., in which the activity varied little between the plant grown in MR and C soil. The highest APX activity was reported in *L. dentata* L. and reached 2.49 U min^−1^ mg^−1^ protein.

Guaiacol peroxidase (GuPX) activity was strongly induced in *O. majorana* L. and *S. officinalis* L. grown on MR (0.33 and 0.17 U min^−1^ mg^−1^ protein, respectively) while it remained unchanged in *R. officinalis* L. with respect to the value measured in control soil (0.087 and 0.091 U min^−1^ mg^−1^ protein, respectively) and it decreased in *L. dentata* L. when compared to the control (0.18 and 0.12 U min^−1^ mg^−1^ protein, respectively). The stimulation of GuPX activity was particularly elevated and greater than that of APX.

### 2.7. Essential Oil Yield and Characterization of Major Compounds

After the 12-week cultivation period, the essential oils were extracted by the hydro-distillation of the aerial parts and the chemical composition was analyzed by gas chromatography and mass spectrophotometry (GC-MS).

The tables present the essential oil composition and yield with the compound retention indices and relative percentage concentrations of *R. officinalis* L. (Table 6), *S. officinalis* L. (Table 7), *O. majorana* L. (Table 8), and *L. dentata* L. (Table 9) grown on phosphate-mine residue (MR) and agriculture soil (C). The percentage composition of various compounds in the essential oil is provided, along with the total percentage and the essential oil yield.

Comparing the essential oil of *R. officinalis* L. composition between C and MR soils, several notable differences are observed. In C soil, the major compounds present in higher percentages include verbenone (17.28%), 1,8-cineole (14.81%), L-borneol (12.88%), camphor (9.77%), α-terpineol (7.12%), linalool (3.15%), terpinen-4-ol (2.92%), and bornyl acetate (2.41%), On the other hand, MR soil exhibits lower percentages of these compounds, with values of 2.12%, 10.81%, 7.89%, 5.06%, 1.84%, and 1.69% for 1,8-cineole, L-borneol, α-terpineol, camphor, terpinen-4-ol, and linalool, respectively. However, in the oil from plants grown on MR soil, the amount of verbenone (the principal component) was almost two times higher than that found in the oil from plants grown on C soil (33.74% and 17.28%, respectively). It is worth noting that MR soil shows higher percentages of certain compounds, such as geraniol (4.86%), *trans*-shisool (2.21%), isoborneol (2.20%), and isoaromadendrene epoxide (1.32%), compared to C soil. Fifty-two constituents were identified, representing about 94.53% of the total oils in C soil, while in MR soil, 42 constituents represented 90.99%. Sixteen constituents disappeared from rosemary essential oil when the plant was grown on MR, while six constituents were detected only in the oil from plants grown on MR. The essential oil yield is found to be comparatively lower in MR soil (1.06% *v/w*; ml 100 g^−1^ dry weight) compared to C soil (1.85%). This indicates a slight reduction in the overall essential oil content when the plant is grown in MR soil.

According to our results, the chemical composition of *R. officinalis* L. essential oil could be organized as follow:

- MR: verbenone > L-borneol > α-terpineol > camphor > geraniol > *trans-*shisool > isoborneol > 1,8-cineole > terpinen-4-ol > linalool.
- C: verbenone > 1,8-cineole > L-borneol > camphor > α-terpineol > linalool > terpinen-4-ol > bornyl acetate.

**Table 6:** Essential oil composition of *R. officinalis* L. grown on phosphate-mine residue and agriculture soil.

Compounds	RI Ref	C		MR	
		RI	%	RI	%
α-pinene	932	912	1.26	887	0.11
Camphene	946	928	0.32	_	_
β-pinene	974	958	0.38	_	_
β-myrcene	988	972	0.36	_	_
δ-2-carene	1001	1001	0.20	_	_
ρ-cymene	1020	1010	0.31	_	_
D-limonene	1024	1014	1.17	998	0.27
1,8-cineole (eucalyptol)	1026	1017	14.81	1002	2.12
Benzeneacetaldehyde	1036	1032	0.24	_	_
γ-terpinene	1054	1048	0.65	_	_
*cis-*sabinene hydrate (IPP vs. OH)	1065	1058	0.63	1046	0.35
ρ-mentha-2,4(8)-diene (terpinolene)	1085	1081	0.62	_	_
Linalool	1095	1093	3.15	1084	1.69
Phenyl ethyl alcohol	1107	1109	0.44	_	_
Chrysanthenone	1124	1123	0.58	1122	0.12
*trans-*pinocarveol	1135	1138	0.28	1129	0.11
*cis-*pinocarveol	1182	_	_	1133	tr
*cis*-verbenol	1137	1140	0.40	1135	0.32
Camphor	1141	1144	9.77	1140	5.06
*cis-*β-terpineol	1140	1148	0.22	_	_
Pinocarvone	1160	1164	1.35	1162	0.59
L-borneol/camphol	1165	1167	12.88	1165	10.81
Terpinen-4-ol	1174	1179	2.92	1178	1.84
ρ-cymen-8-ol	1179	_	_	1186	0.74
α-terpineol	1186	1193	7.12	1193	7.89
Myrtenol	1194	1200	0.56	1200	0.61
Isoborneol	1155	_	_	1206	2.20
Verbenone	1204	1214	17.28	1214	33.74
2-Oxabicyclo [2.2.2] octan-6-ol, 1,3,3-trimethyl-	_	_	_	1220	0.51
*cis-*carveol	1226	1223	0.18	1223	0.41
β-citronellol	1223	1231	0.48	1231	0.49
*trans*-shisool	1248	1248	1.65	1248	2.21
Geraniol	1249	1258	1.34	1258	4.86
Geranial	1264	1275	0.31	_	_
Isopiperitenone	1272	1278	0.29	1278	0.89
Bornyl acetate	1284	1291	2.41	_	_
Acetic acid, 1,7,7-trimethyl-bicyclo [2.2.1] hept-2-yl ester	1277	_	_	1292	0.90
Carvacrol	1298	1300	0.19	1306	0.39
Piperitenone	1340	1347	0.45	1347	1.06
Eugenol	1356	1361	0.40	1361	0.78
α-copaene	1374	1380	0.35	_	_
Methyleugenol	1403	1405	0.50	1405	0.74
β-caryophyllene	1417	1422	1.65	1422	0.35
α-humulene	1452	1453	1.36	1453	0.46
γ -muurolene	1478	1473	0.25	_	_
epi-cubebol	1493	1489	0.24	1473	0.05
Butylated hydroxytoluene	1514	1504	0.64	1504	1.71
γ -cadinene	1513	1509	0.36	_	_
δ-cadinene	1522	1518	0.58	1519	0.33
Caryophyllene oxide	1582	1589	0.75	1589	0.78
Humulene epoxide II	1608	1618	0.44	1618	0.68
Humulenol II	1632	1642	0.25	1643	0.71
Caryophylla-4(12),8(13)-dien-5α-ol	1639	_	_	1647	0.59
α-epi-cadinol	1640	1650	0.20	1651	0.59
2-(7-Heptadecynyloxy) tetrahydro-2H-pyran	_	1655	0.23	_	_
α-cadinol	1652	1665	0.18	1665	0.63
Isoaromadendrene epoxide	_	1669	0.46	1669	1.32
α-bisabolol	1685	1693	0.47	1693	0.90
TOTAL			94.53		90.99
EO yield (%)			1.85		1.06

C: Agriculture soil; MR: mine residue; RI: retention index; EO: essential oil.

The hydrodistillation of the aerial parts of an *S. officinalis* L. plants grown on MR and C soil gave yellow oils in 2.88% and 1.996% yields, respectively. The essential oil content increased by almost 44% because of the effect of changing the growth medium. The principal components of both oils were identified as 1,8-cineole (6.24% in the oil from the plants grown on C soil and 2.81% in the oil from plants grown on MR), α-thujone (16.18%; 11.00%), D-camphor (6.43%; 6.85%), β-caryophyllene (14.04%; 4.88%), α-humulene (10.38%; 4.38%), viridiflorol (10.31%; 14.10%), and 13-epi-manool (15.07%; 22.22%). MR soil shows higher percentages of derivates of some compounds, such as caryophyllene oxide (4.32%) and humulene epoxide II (5.11%). Sixty-four constituents were identified, representing 95.53% of the total oils in C soil. Essential oil from the plants grown on MR had 14 constituents less than the oil from plants grown on C soil and the identified constituents represent 94.21% of the total oils in MR.

The main constituents of *S. officinalis* L. essential oil could be divided into two groups, as follows:

- MR: 13-epi-manool > viridiflorol > α-thujone > D-camphor > jhumulene epoxide II > β-caryophyllene > α-humulene > caryophyllene oxide > 1,8-cineole.
- C: α-thujone > 13-epi-manool > β-caryophyllene > α-humulene > viridiflorol > D-camphor > 1,8-cineole.

**Table 7:** Essential oil composition of *S. officinalis* L. grown on phosphate-mine residue and agriculture soil.

Compounds	RI Ref	C		MR	
		RI	%	RI	%
α-thujene	924	880	tr	_	_
α-pinene	932	887	0.23	887	0.14
Camphene	946	905	0.24	905	0.11
L-β-pinene	974	937	0.47	937	0.12
β-myrcene	988	953	0.31	953	0.11
3-octanol	988	959	0.13	_	_
δ-2-carene	1001	984	tr	_	_
ρ-cymene	1020	994	0.12	994	Tr
D-limonene	1024	999	0.25	999	0.15
1,8-cineole	1026	1002	6.24	1002	2.81
γ-terpinene	1054	1035	0.23	_	_
*cis-*sabinene hydrate (IPP vs. OH)	1065	1046	0.19	1047	0.22
Terpinolene	1085	1071	0.11	_	_
*trans*-sabinene hydrate (IPP vs. OH)	1098	1083	0.29	1084	0.12
β-thujone	1112	1092	1.97	1093	1.68
α-thujone	1101	1105	16.18	1105	11.00
Iso-3-thujanol	1134	1126	0.17	1126	0.45
D-camphor	1141	1140	6.43	1140	6.85
*trans*-pinocamphone	1158	1159	0.15	1159	tr
L-borneol	1165	1165	0.78	1165	0.98
*cis*-pinocamphone	1172	1175	0.16	1176	tr
Terpinen-4-ol	1174	1178	0.56	1178	0.44
4-hydroxy-α-thujone	1213	1190	0.13	1190	0.51
α-terpineol	1186	1193	0.11	1194	tr
4-hydroxy-β-thujone	1213	1201	0.64	1201	1.16
2-hydroxy-1,8-cineole	1247	1215	0.18	_	_
ρ-mentha-1(7),8(10)-dien-9-ol	1256	1230	0.14	_	_
Bornyl acetate	1284	1292	0.29	1292	0.65
ρ-cymen-7-ol	1289	1296	0.15	1297	0.55
Carvacrol	1298	1306	0.16	1306	0.33
Eugenol	1356	1361	0.10	_	_
α-copaene	1374	1380	0.15	1380	tr
β-caryophyllene	1417	1422	14.04	1422	4.88
β-copaene	1430	1430	0.24	1430	0.07
α-panasinsene	1416	1436	0.14	_	_
Aromadendrene	1439	1440	0.72	1440	0.21
Selina-5,11-diene	1474	1444	0.12	_	_
α-humulene	1454	1453	10.38	1453	4.38
Alloaromadendrene	1458	1460	0.21	1460	0.14
γ-muurolene	1478	1473	0.32	1473	0.60
β-selinene	1489	1483	Tr	1483	0.33
Viridiflorene	1496	1490	0.57	1490	tr
α-muurolene	1500	1494	Tr	1494	tr
Butylated hydroxytoluene	1514	1504	0.31	1503	0.76
γ-cadinene	1513	1509	0.21	1507	0.41
δ-cadinene	1522	1519	0.50	1514	0.75
α-calacorene	1544	1542	Tr	1542	0.34
Spathulenol	1577	1582	0.14	1582	tr
Caryophyllene oxide	1582	1589	0.96	1590	4.32
Viridiflorol	1592	1599	10.31	1599	14.10
Humulene epoxide I	1592	1607	0.24	1607	0.79
Humulene epoxide II	1608	1618	0.92	1618	5.11
Humulenol II	1632	1642	0.21	1643	1.48
Caryophylla-4(12),8(13)-dien-5α-ol	1639	1647	0.13	1647	0.64
α-epi-muurolol	1640	1666	0.11	1665	0.17
14-hydroxy-9-epi-(E)-caryophyllene	1668	1669	0.17	1669	0.93
Germacra-4(15),5,10(14)-trien-1-α-ol	1685	1683	0.32	1683	1.17
14-hydroxy-αhumulene	1713	1728	0.14	_	_
Isopimara-9(11),15-diene	1905	1928	0.17	_	_
13-epi-manool	2059	2075	15.07	2075	22.22
Labda-7,14-dien-13(R)-ol	2096	2106	0.91	2106	1.57
Sclareol	2222	2256	0.10	_	_
*trans*-ferruginol	2331	2357	0.45	_	_
TOTAL			95.53		94.21
EO yield (%)			1.996		2.88

C: Agriculture soil; MR: mine residue; RI: retention index; EO: essential oil.

Analysis of the chemical composition of *O. majorana* L. essential oils revealed the presence of 22 components in C soil (96.47% of total oil) and 18 components in MR (97.22% of total oil) represented mainly by *cis*-sabinene hydrate (IPP vs. OH) (67.44% in the oil from the plants grown on C soil and 52.32% in the oil from plants grown on MR), trans-sabinene hydrate (IPP vs. OH) (3.96%; 3.24%), terpinen-4-ol (8.00%; 9.93%), L-α-terpineol (8.75%; 8.56%), linalyl acetate (1.35%; 2.07%), and butylated hydroxytoluene (1.757%; 12.46%). Plants grown on MR show a higher percentage (>1%) of other components compared to plants grown in the control soil, such as L-borneol (1.22%), raspberry ketone methyl ether (1.12%), spathulenol (1.23%), and caryophyllene oxide (1.92%). One component was detected only in the oil from plants grown on MR, namely bornyl acetate, with a low percentage (0.24%). The essential oil content was higher in *O. majorana* L. grown on MR with a yield of 3.06%, while in C soil the yield was 2.48%.

According to the amounts of the major constituents, *O. majorana* L. essential oil could be divided into two groups, as follows:

- MR: *cis*-sabinene hydrate > butylated hydroxytoluene > terpinen-4-ol > L-α-terpineol > *trans*-sabinene hydrate > linalyl acetate.
- C: *cis*-sabinene hydrate > L-α-terpineol > terpinen-4-ol > *trans*-sabinene hydrate > butylated hydroxytoluene > linalyl acetate.

**Table 8:** Essential oil composition of *O. majorana* L. grown on phosphate-mine residue and agriculture soil.

Compounds	RI Ref	C		MR	
		RI	%	RI	%
1,8-cineole	1026	1002	0.30	1003	0.23
γ-terpinene	1054	1035	0.35	_	_
*trans*-sabinene hydrate (IPP vs. OH)	1098	1045	3.96	1046	3.24
*cis*-sabinene hydrate (IPP vs. OH)	1065	1082	67.44	1083	52.32
*cis*-ρ-menth-2-en-1-ol	1118	1110	0.70	1111	0.91
*trans*-ρ-menth-2-en-1-ol	1136	1132	0.27	1132	0.36
D-camphor	1141	1140	0.22	1140	0.68
L-borneol	1165	1165	0.22	1165	1.22
Terpinen-4-ol	1174	1177	8.00	1177	9.93
L-α-terpineol	1186	1192	8.75	1193	8.56
*cis*-piperitol	1195	1198	0.34	1198	0.17
*trans-*dihydro carvone	1200	1201	0.30	_	_
*trans-*piperitol	1207	1211	0.38	1211	0.20
*cis-*ρ-mentha-1(7),8-dien-2-ol	1227	1222	0.09	_	_
*trans-*chrysanthenyl acetat	1235	1226	0.15	_	_
*cis-*myrtanol	1250	1231	0.13	_	_
Linalyl acetate	1254	1258	1.35	1258	2.07
Bornyl acetate	1284	_	_	1292	0.24
Geranyl acetate	1379	1383	0.31	1384	0.37
Raspberry ketone methyl ether	1493	1492	0.30	1492	1.12
Butylated hydroxytoluene	1514	1504	1.77	1504	12.46
Spathulenol	1577	1582	0.46	1582	1.23
Caryophyllene oxide	1582	1589	0.66	1589	1.92
TOTAL			96.47		97.22
EO yield (%)			2.48		3.06

C: Agriculture soil; MR: mine residue; RI: retention index; EO: essential oil.

The main constituents of the essential oil of *L. dentata* L. grown on C soil were camphor (27.33%), 1,8-cineole (16.94%), fenchone (3.60%), borneol (3.01%), *trans*-pinocarveol (2.97%), myrtenol (2.88%), linalool (2.57%), β-eudesmol (2.42%), and *cis*-14-nor-muurol-5-en-4-one (1.99%). The percentage of these components changed when the plants were grown on MR, with values of 20.61% for camphor and 12.26%, 5.54%, 4.41%, 3.89%, 3.71%, 2.71%, 2.61%, 2.36%, 2.28%, and 2.26% for 1,8-cineole, butylated hydroxytoluene, coumarin, β-eudesmol, *cis*-14-nor-muurol-5-en-4-one, myrtenol, fenchone, linalool, borneol, and trans-pinocarveol, respectively. *L. dentata* L. essential oil was rich in constituents. A total of 51 components were identified, representing 86.16% of the total oil in C soil, while 34 components were detected and identified in oil from plants grown on MR, representing 84.14% of the total oil. Hydrodistillation of *L. dentata* L. leaves provided a yield of about 1.112% when it was grown in C soil, while it offered a higher yield when grown in MR (1.8%).

The main constituents of *L. dentata* L. essential oil could be organized as follows:

- MR: 1,8-cineole > butylated hydroxytoluene > coumarin > β-eudesmol > *cis-*14-nor-muurol-5-en-4-one > myrtenol > fenchone > linalool > borneol > *trans-*pinocarveol.
- C: camphor > 1,8-cineole > fenchone > borneol > *trans-*pinocarveol > myrtenol > linalool > β-eudesmol > *cis-*14-nor-muurol-5-en-4-one.

**Table 9:** Essential oil composition of *L. dentata* L. grown on phosphate-mine residue and agriculture soil.

Compounds	RI Ref	C		MR	
		RI	%	RI	%
α-pinene	932	887	0.14	_	_
Camphene	946	904	0.12	_	_
β-pinene	974	937	0.41	_	_
Dehydro-1,8-cineol	988	954	0.23	_	_
D-limonene	1024	998	0.14	_	_
1,8-cineole (eucalyptol)	1026	1002	16.94	1002	12.26
o-cresol	1050	1030	0.30	_	_
*trans-*sabinene hydrate (4-thujanol)	1098	1045	0.16	1046	0.22
Linalool oxide B	1067	1051	1.86	1052	1.28
Fenchone	1083	1071	3.60	1072	2.61
Linalool	1095	1083	2.57	1084	2.36
α-fenchol	1114	1102	1.10	1102	0.74
Dehydro-sabina ketone	1117	1110	0.27	_	_
α-campholenal	1122	1117	0.27	_	_
4-Acetyl-1-methylcyclohexene	_	1123	0.16	_	_
*trans-*pinocarveol	1135	1132	2.97	1132	2.26
*cis-*verbenol	1137	1135	0.25	_	_
Camphor	1141	1139	27.33	1140	20.61
Sabina ketone	1154	1155	0.16	_	_
Pinocarvone	1160	1161	0.98	1161	0.98
Borneol	1165	1165	3.01	1165	2.28
Linalool oxide D	1170	1167	0.60	1168	0.64
Linalool oxide C	1173	1173	0.63	1173	0.73
Terpinen-4-ol	1174	1177	0.54	1178	0.57
ρ-cymen-8-ol	1179	1186	0.76	1186	1.22
*trans-* ρ-mentha-1(7),8-dien-2-ol	1187	1189	0.14	_	_
α-terpineol	1186	1193	1.27	1193	1.49
Myrtenol	1194	1200	2.88	1201	2.71
L-verbenone	1204	1214	0.74	1215	0.92
*cis-*carveol	1226	1223	0.55	1223	0.76
D-carvone	1239	1249	0.34	1249	0.28
1,3-cyclohexadiene-1-methanol, 4-(1-methylethyl)-	_	1286	0.13	_	_
ρ-cymen-7-ol	1289	1295	0.28	1296	0.77
Perilla alcohol	1294	1304	0.47	1304	0.70
Dodecane, 2,6,11-trimethyl	1320	_	_	1327	0.62
ρ-mentha-1,4-dien-7-ol	1325	_	_	1333	0.74
1-tetradecene	1388	_	_	1389	0.54
α-*trans*-bergamotene	1432	1434	0.19	_	_
Coumarin	1432	1438	0.57	1438	4.41
β-selinene	1489	1483	1.33	_	_
β-bisabolene	1505	1499	0.33	_	_
Butylated hydroxytoluene	1514	1504	0.40	1504	5.54
γ-cadinene	1513	1509	0.34	_	_
*trans-*calamenene	1521	1519	0.66	_	_
Naphthalene, 1,2,3,4-tetrahydro-2,5,8-trimethyl-	_	1543	0.84	1544	1.72
*trans-*sesquisabinene hydrate	1577	1580	0.11	_	_
Caryophyllene oxide	1582	1589	1.72	1589	1.31
1,10-di-epi-cubenol	1618	1623	0.26	_	_
β-eudesmol	1649	1662	2.42	1662	3.89
α-bisabolol oxide B	1656	1664	0.87	1665	1.38
Cadalene	1675	1687	0.54	_	_
*trans-*10-hydroxycalamenene	1687	_	_	1680	1.02
α-bisabolol	1685	1693	0.87	1693	1.17
*cis-*14-nor-muurol-5-en-4-one	1688	1703	1.99	1703	3.71
Murolan-3,9(11)-diene-10-peroxy	1729	1733	0.45	1733	1.71
TOTAL			86.18		84.14
EO yield (%)			1.112		1.8

C: Agriculture soil; MR: mine residue; RI: retention index; EO: essential oil.

The major differences between the oils from the plants grown on MR and C soil were observed mainly in the number of constituents detected, the percentage of the main components, and the essential oils yields. The variations in essential oil composition and yield between C and MR soils can be attributed to several factors, including differences in soil nutrient availability, environmental conditions, and genetic factors. Soil composition plays a crucial role in the synthesis of specific compounds in plants, and the observed differences may reflect the adaptability of the four aromatic plants to different soil conditions.

## 3. Discussion

This investigative study revealed that utilizing the pure phosphate-mine residue as a growth substrate did not promote optimal plant growth and development compared to the agriculture soil used as a control soil (Figure 1 and Table 3). *S. officinalis* L., *L. dentata* L., and *O. majorana* L. showed the poorest growth on MR soil compared to their respective controls. However, planted *R. officinalis* L. seedlings were unaffected by growth media. This outcome can be attributed not only to chemically adverse conditions that undermine plant viability and growth [37] but also to the unfavorable physical attributes and structural qualities of the residue itself, which collectively contribute to a reduced plant survival rate [38]. The lower SFW and SDW and the decrease in SL observed on the plants grown in phosphate-mine residues suggest the presence of growth-limiting factors or reduced nutrient availability in these soils (Table 3). These findings align with previous studies indicating the adverse effects of mine residues on plant growth due to altered soil characteristics and limited nutrient content [39,40,41].

Growing a plant on infertile or degraded soil can be considered as a stress for the plant [42]. This is because plants require a range of essential nutrients and minerals from the soil to grow and thrive. When the soil is infertile or degraded, it lacks these necessary nutrients, and the plant may struggle to obtain what it needs to grow properly [43]. Despite the sufficient availability of essential plant nutrients within the soil, the assimilation of these nutrients by plant roots is not always assured. An illustrative scenario of this phenomenon arises in compacted soils characterized by insufficient aeration. In such instances, inadequate oxygen levels in the rhizosphere can inhibit the active uptake of nutrients by the roots themselves [44,45]. In this study, it was found that the plants grown in C soil exhibited high performance in terms of root development and growth compared to those in MR (Figure 2 and Table 4). The variations in root parameters among the plant species suggest species-specific responses to soil type. *R. officinalis* L. appeared to be particularly well-suited to thrive in MR soil with comparable root development to growth with C soil. *L. dentata* L. exhibited a decrease in most parameters. *O. majorana* L. and *S. officinalis* L. also experienced negative effects on root parameters in MR, albeit to a lesser extent. C soil generally promotes higher values of root mass, length, surface area, volume, and, in some cases, average diameter, indicating enhanced root development and nutrient uptake potential. These findings highlight the adverse impact of MR soil structure on root characteristics and plant performance across the studied species. Plant cultivation on MR restricted root growth, nutrient absorption, and water uptake, ultimately affecting the overall vigor and health of the plants. In compacted soils, such as the mine residue used in this study, the reduction in pore space, lack of soil aeration, and water infiltration limits root growth and penetration, leading to shorter roots and reduced surface area [46,47]. Conversely, in degraded soils, the reduction in nutrient availability and quality can result in shorter roots with reduced surface area as well [48]. Root diameter and volume, on the other hand, are less likely to be directly impacted by soil compaction and degradation. However, the reduction in root length and surface area can indirectly affect root diameter and volume by limiting nutrient and water uptake, which can result in smaller and less-developed roots [46]. It is important to consider these negative effects when assessing the suitability of MR environments for plant growth and to develop appropriate strategies for reclamation and restoration efforts.

A deviation in chlorophyll content is generally related to the plant stress; the synthesis and accumulation of chlorophylls increased in response to low-level stress and decreased in response to high-level stress [49]. Chlorophyll molecules exist in numerous forms, the most prevalent of which are *a* and *b*. The concentrations of chlorophyll *a* and *b* depend on several factors, such as nutrient deficiency, light exposure, temperature, and abiotic stressors. According to the studies of many researchers, the chlorophyll content in plants decreased under saline conditions [50,51]. Under nutritional stress, the changes in nitrogen metabolism in relation to the formation of substances, such as proline, which is involved in osmotic control, may also contribute to a decreased chlorophyll concentration [52]. According to Negi et al. [53], N stress damages the internal chloroplast structure and decreases the chlorophyll concentration, resulting in plants being subject to light damage. In the current study, the content of chlorophyll *a* was higher in the leaves of *L. dentata* L., *O. majorana* L., and *S. officinalis* L. under the C condition and was decreased when the plants were grown in MR soil. A similar trend was observed with *c*hlorophyll *b.* Similar findings were reported by Kulbat Warycha et al. [23]. On the other hand, carotenoids serve as efficient antioxidants and supplementary pigments to preserve and stabilize photochemical processes [54]. A decrease in carotenoids content in *O. majorana* L. and *S. officinalis* L. grown on MR soil demonstrates the vulnerability of those plants’ photosynthetic systems to stress. In contrast to what was previously mentioned, there was an increase in carotenoid content in *L. dentata* L. and *R. officinalis* L. grown on MR, which clearly demonstrated its protective function against oxidative damage [55,56]. The studied plants probably have diverse methods of photosynthetic apparatus response to stress action. A reduction in carotenoid concentration under N deficiency has been shown by Maswada et al. [57], despite the fact that carotenoids are N-free. The reason is that low N may inhibit carotenoid formation via the dimerization of geranylgeranyl pyrophosphate [58].

In response to this stress, plants may activate various physiological and biochemical mechanisms to help them adapt and survive. For example, they may produce compounds that help them tolerate the specific stresses associated with infertile or degraded soils. However, if the stress is too severe or prolonged, the plant may not be able to cope, and its growth and yield may be severely compromised. In stressed plants, there are complex relationships and interactions between various biochemical components, including the accumulation of osmolytes (proline and soluble sugars), proteins, polyphenols, flavonoids, and MDA (malondialdehyde).

Osmolyte generation is a common occurence in stress reduction for minimizing physiological damage. Under stress conditions, increased levels of proline are often correlated with enhanced tolerance and adaptation. Proline can scavenge reactive oxygen species (ROS) and stabilize cellular structures, thereby reducing oxidative stress and lipid peroxidation, as indicated by lower MDA levels. The outcomes of our experiment suggest that the concentration of proline in *L. dentata* L., *R. officinalis* L.,*O. majorana* L., and *S. officinalis* L. cultivated in agricultural soil is higher than that of the ones grown in mine residue (Figure 3). This difference implies that plants exhibit reduced tolerance when cultivated in the mine residue as a substrate. It is possible that the lower concentration of proline in plants grown in mine residue could be due to the nutrient deficiencies in the soil, which may have led to oxidative stress and reduced proline synthesis in the plant. Alternatively, the differences in proline content could be due to other factors and environmental conditions. Nutrient deficiencies in the mine residue, such as nitrogen, phosphorus, and potassium, can disrupt normal plant metabolism, including proline biosynthesis [59]. Extreme alkaline pH values and the absence of adequate organic matter in MR can similarly affect nutrient availability and uptake by plants. Proline content in plants decreases when proline synthesis is inhibited, or when proline degradation is enhanced. The inhibition of proline synthesis may occur under conditions of N deficiency; since proline is derived from glutamate, which requires nitrogen as a precursor, a deficiency in nitrogen can limit the availability of nitrogenous substrates for proline metabolism and, subsequently, reduce proline synthesis [60]. On the other hand, the plant under N stress would need to degrade proline, which is utilized as a nitrogenous source for the synthesis of other amino acids [61]. Soluble sugars and soluble proteins accumulation is also an adaptive response to stress conditions. Soluble sugars, such as glucose and sucrose, serve as a source of energy and as osmo-protectants, helping plants maintain turgor and cellular water balance during stress [62]. Soluble proteins are also important osmotic regulatory substances. Soluble sugars can also act as antioxidants, directly scavenging ROS [63,64]. Additionally, soluble sugars can indirectly protect the plant from ROS by maintaining the balance of other antioxidant systems in the plant, such as the ascorbate–glutathione cycle [65]. The accumulation of soluble sugars and soluble proteins during stress is associated with increased stress tolerance and enhanced protection against oxidative damage [62,66,67]. In this study, soluble sugars were enhanced in plants grown on MR compared to the control, except for *O. majorana* L., where the abundance of soluble sugars was significantly lower in plants grown in MR than those grown in C soil. The increased synthesis of soluble sugars under stress might be attributed to the hydrolysis of sucrose and starch increasing the concentration of glucose and fructose [68]. High accumulation of soluble proteins was also reported in *L. dentata* L. and *S. officinalis* L. plants grown on MR compared to the control plants.

Polyphenols and flavonoids are important groups of plant secondary metabolites with strong antioxidant properties and protective functions under stressful environmental conditions [9,69,70]. These compounds can scavenge ROS and prevent oxidative damage to cellular components. Under stress conditions, plants often increase the production of polyphenols and flavonoids as part of their adaptive response [71,72]. Higher levels of polyphenols and flavonoids are associated with improved antioxidant capacity, reduced lipid peroxidation (lower MDA levels), and enhanced stress tolerance. In general, moderate stress can stimulate the production of polyphenols, while severe stress can cause a decrease in polyphenol content [30,72]. The polyphenol and flavonoid contents were not affected in *R. officinalis* L. plants, while only flavonoids were decreased in *L. dentata* L., plant grown on MR. An increase in polyphenols and a decline in flavonoid content in *S. officinalis* L. cultivated on MR was observed; however, an inhibition of both polyphenols and flavonoids was noted in *O majorana* L. grown on MR. The variations in polyphenols and flavonoids content between plants grown on C and MR soils could indicate differences in the stress levels experienced by the plants in each soil type. Those variations among the studied plant species may be attributed to several factors, including genetic differences, environmental conditions, and soil composition. Jakovljević et al. [71] show that nutrient concentrations influence the production of these compounds; hence, the quantity of bioactive compounds will be influenced by the nature and quantity of nutrients, the type of cultivar, and the plant part used for extraction. Previous research reported an inhibition of phenolic production in response to Cd stress at high concentration (at the level of 4 mg L^−1^) in mangrove plants [72]. The reason for enhanced phenolic compounds in *S. officinalis *L. grown on MR might link to increased soluble sugars in this plant, as soluble sugars and related metabolites from the tricarboxylic acid cycle might function as substrates for the subsequent production of polyphenol compounds via the shikimic acid pathway [73].

The present results regarding MDA revealed that there is an increase in MDA production in plants grown on MR soil except for *R. officinalis* L. (Figure 3). Elevated MDA levels in stressed plants indicate the extent of oxidative damage to cellular membranes. It serves as an indirect measure of the severity of oxidative stress and can be used as a biochemical marker to assess the degree of stress experienced by plants. Higher MDA levels are indicative of more significant oxidative damage, while lower levels suggest a better antioxidant defense system or less severe stress conditions. Our data are in agreement with Mansori et al. [74] who demonstrated a significant increase in MDA content in the leaves of *S. officinalis* L. under water stress, suggesting that membrane lipid peroxidation could be induced by water deficit. The same trend has been demonstrated on *Origanum vulgare* L. [75] under water stress. In contrast to our results, *R. officinalis* L. exposed to salt stress in a previous report exhibited a high accumulation of MDA [76]. However, the authors demonstrate that MDA accumulation does not necessarily indicate oxidative damage in leaves because *R. officinalis* L. plants were able to tolerate this elevated oxidative stress, as evidenced by the maintenance of plant development and even better water contents and chlorophyll levels [76]. The observed increase in lipid peroxidation in the leaves of *L. dentata* L., *O. majorana* L., and *S. officinalis* L. plants grown on MR may be partly responsible for the decrease in photosynthetic pigments in those plants. On the other hand, since the seedlings were grown on MR under prolonged conditions of stress and nutrient limitation, a high MDA level can indicate significant oxidative damage and may be associated with lower polyphenolic and flavonoid contents and lower antioxidant activity.

Contrary to control plants, an increase in the APX activity was recorded in all plants grown on MR; however, the GuPX activity was increased in only *S. officinalis* L. and *O. majorana* L. when grown on MR. *L. dentata* L. grown on MR exhibited lower GuPX activity compared to the control plant, while there is no significant variation in the GuPX activity in *R. officinalis* L. Antioxidant enzymes are essential for protecting plants from various stresses. They mitigate the harmful effects of reactive oxygen species (ROS) generated during abiotic stresses, like exposure to pesticides, heavy metals, drought, and salinity [77,78]. These enzymes help maintain cellular balance and prevent oxidative damage. In previous studies, *R. officinalis* L. under salt stress showed an increase in GuPX activity [79] and APX activity [80].

In our study, *R. officinalis* L. was able to tolerate oxidative stress, as seen via the maintenance of plant development, better chlorophyll levels, and even by the unchanged values between the control plant and the plants grown on MR for some biochemical compounds involved in antioxidative protection, indicating that *R. officinalis* L. was not under stress when grown on MR. The reduced biomass in the other plants grown on MR could be due to the failure of the antioxidative enzymes to capture ROS.

Essential oils from the leaves of plant grown under control or MR soil conditions were analyzed by GC-MS to better understand the variations in essential oil (EO) content and composition in response to growth medium variation and nutrient deficiency stress. The composition of EOs is affected by many factors, such as climatic factors [80], genetic factors, developmental stage, the type of plant material (flowers, leaves, leaves, stem, etc.), and the extraction method [81]. Environmental conditions also affect the quantity and quality of EOs, with factors, such as nutrient deficiency, drought, salinity, or HMs, having an impact [82]. Major constituents of essential oils are also modified under several factors.

The essential oil of *R. officinalis* L. is known for its various pharmacological and therapeutic properties, including its antimicrobial, antioxidant, and anti-inflammatory effects. Comparing the results of *R. officinalis* L. grown on MR in this study with findings from other studies provides insights into the response of the plant to different soil conditions. Several compounds in the essential oil composition exhibit variations when *R. officinalis* L. is cultivated on MR compared to in control soil, indicating potential adaptations of the plant to these challenging environments. Our results indicate a slight reduction in the overall essential oil content when the *R. officinalis* L. plant is grown in MR soil. Previous research has reported a significantly decreased essential oil yield for *R. officinalis* L. under water stress, which is mainly due to the decreased photosynthesis process [83].

In terms of major compounds, our study shows that verbenone, 1,8-cineole, L-borneol/camphol, camphor, and α-terpineol are present in notable percentages in *R. officinalis* L grown on control soil. Tounekti et al. (2011) [84] have reported previously a similar EO profile with verbenone as a major component. Previous studies have reported 1,8-cineole [85] or camphor [12] as predominant constituents of *R. officinalis* L. essential oil. However, when *R. officinalis* L. is grown on MR, the percentages of these major compounds tend to decrease, except for verbenone, which increased. Similar observations have been made in other studies investigating *R. officinalis* L. cultivation under stress, indicating the potential influence of stress on the synthesis of these compounds [80]. Overall, the findings from this study align with previous research, indicating that the essential oil composition of *R. officinalis* L. can be influenced by soil degradation and stress. Soil fertility could also affect the production, release, or storage of essential oil through modulating the plant’s nutritional status, which subsequently controls its biochemical composition [86].

The essential oil content of *S. officinalis* L. plants grown on MR increased by almost 44% compared to the control plant. The results showed that all identified compounds were significantly affected by the stress caused by the MR soil. The principal component of the EO from the control plant was α-thujone. The same main constituent was identified in *S. officinalis* L. in a previous study [10,12]. A different profile was described by El Euch et al. (2019) [34] where Camphor is the main constituent of the studied *S. officinalis* L. EO. The main constituent of *S. officinalis* L. essential oil grown on MR was 13-epi-manool (22.22%), which was more abundant than in plants grown in C soil (15.07%). The comparison between the 2 sets of essential oils revealed that while 64 constituents were identified in oils from C soil, the essential oil from MR-grown plants contained 14 fewer constituents, representing 94.21% of the total oils.

The essential oil content was found to be notably higher in *O. majorana* L. cultivated in MR (yielding 3.06%) compared to C soil (yielding 2.48%). The same trend was observed in *O. majorana* L. under moderate salinity [87]. The findings indicated the presence of 22 components in control plant EO, constituting 96.47% of the total oil, while 18 components were identified in plants grown on MR, comprising 97.22% of the total oil. The primary constituents in both soils were *cis*-sabinene hydrate (IPP vs. OH) and *trans*-sabinene hydrate (IPP vs. OH), but the proportions varied significantly between the two soils. Notably, *cis*-sabinene hydrate (IPP vs. OH) was the predominant component in plants using both soil types, accounting for 67.44% in C soil-grown plants and for 52.32% in MR-grown plants. Our results are in agreement with other research where *cis*-sabinene hydrate (IPP vs. OH) (reported with the old name *trans*-Sabinene hydrate) was reported as the main constituent of the EO of a Tunisian variety of *O. majorana* L. [87]. Other studies report terpinen-4-ol as main constituent of *O. majorana* L. EO [88].

The yield of essential oil from *L. dentata* L. demonstrated a notable difference, with plants grown in MR offering a higher yield of approximately 1.8% compared to 1.112% from C soil-grown plants. This significant variance in yield further underscores the impact of soil type on the production of essential oils in *L. dentata* L. In oils extracted from *L. dentata* L. grown on C soil, camphor emerged as the principal constituent, representing 27.33% of the total oil. However, when the same plants were cultivated in MR, there were notable shifts in the percentage of this component. Camphor decreased to 20.61%, and the proportions of other constituents, such as 1,8-cineole, butylated hydroxytoluene, coumarin, and several others, showed variations compared to their concentrations in C soil. This study’s findings align with prior research emphasizing that camphor was the major compound (50.3%) of *L. dentata* L. EO followed by other components with relatively small amounts [89]. The comparison between the oils from C soil and MR exhibited differences in both the number and proportion of identified constituents. The oil from C soil contained 51 identified components, representing 86.16% of the total oil, while the oil from MR contained 34 components, representing 84.14% of the total oil. This discrepancy suggests qualitative differences in the chemical profile of the oils due to the influence of soil type on the plant secondary metabolites.

This research underscores the substantial influence of soil variation on the yield and chemical composition of the studied plant essential oils. The substantial variations in constituent proportions and yield between C soil and MR provide valuable insights into the impact of environmental factors on the qualitative and quantitative aspects of essential oils, advocating for further investigation into the role of soil conditions in shaping plant secondary metabolites. It is important to choose plant species and cultivars that are adapted to the specific soil conditions and to employ appropriate soil management practices to improve soil fertility and health.

Although this study provides valuable insights into the growth, biochemical responses, and essential oil composition of *R. officinalis* L., *S. officinalis* L.*, L. dentata* L.*,* and *O. majorana* L. grown in phosphate-mine residue, we acknowledge the limitation posed by the relatively small number of biological replicates (five replicates). While this may affect our ability to capture the full extent of variability among plants, the trends observed offer important preliminary information. Further research, with larger sample sizes, is necessary to validate these findings and fully explore the impact of mine residue conditions on plant performance and essential oil quality.

## 4. Materials and Methods

### 4.1. Experimental Setup and Plant Growth Conditions

The experiments were conducted under greenhouse conditions at the Agricultural Innovation and Technology Transfer Center, Mohammed VI Polytechnic University (AITTC-UM6P), Benguerir, Morocco (32°13′11.3″ N, 7°53′31.8″ W), from November 2021 to February 2022. The experimental setup used a completely randomized design.

Seedlings of uniform length (10 to 15 cm heigh) of *R. officinalis* L., *S. officinalis* L., *L. dentata* L., and *O. majorana* L. were transferred to plastic pots containing 2 kg of each of the two substrates used, namely a control soil (agriculture soil collected from the experimental farm at AITTC) and a phosphate-mine residue (sterile waste) sampled from the Benguerir experimental mine site belonging to OCP (32°12′33.5″ N, 7°49′38.8″ W), where they were grown for 16 weeks. The Benguerir experimental mine site had been previously mined for rock phosphate and used as a dump for material after phosphate extraction (called mine residue, MR). Three seedling per pot were planted and replicated 6 times for the control soil and mine residue (resulting in 18 seedlings of the tested plants/tested soil) under natural conditions of light and average night and day temperatures of 9.5 °C and 24.5 °C, respectively (minimum 5 °C and maximum 30 °C). Irrigation of the pots to 70% field capacity was conducted on a daily basis.

### 4.2. Soil Analysis

In order to determine the physicochemical properties of phosphate-mine residue and the control soil, several subsamples were randomly collected from a depth of 0–15 cm and mixed to prepare a composite sample. Each of the collected samples was air dried and sieved to pass through a 2 mm mesh size. Soil pH and electrical conductivity were measured in a 1:5 ratio with an InLab Pure Pro-ISM pH electrode (Mettler Toledo, Columbus, OH, USA) and an InLab 741-ISM conductive electrode (Mettler Toledo, Columbus, OH, USA), respectively, using a SevenExcellence™ pH/mV meter according to the NF ISO 10390 [90] and NF ISO 11265 [91] standards, respectively. Total organic carbon (TOC) was measured using the loss on ignition method [92,93] where the samples were calcinated at 650 °C in a muffle furnace for 3 h (Nabertherm GmbH Bahnhofstr. 20, 28865 Lilienthal, Bremen, Germany), and available phosphorous (P) were examined by the method of Olsen et al. [94] according to the NM ISO 11263 [95] standard. All the analyses were performed in five replicates.

Total nitrogen, exchangeable potassium, and soil texture were analyzed by the Agricultural Innovation and Technology Transfer Center laboratories. Total nitrogen was determined using Kjeldahl method [96]. Exchangeable potassium was analyzed by atomic absorption spectroscopy after ammonium acetate extraction according to the NF X 31-108 [97] standard. Soil texture was determined by Robinson’s pipette method according to the NF X 31-107 [98] standard.

### 4.3. Plant Harvest and Preparation for Analysis

Shoot length was measured, and the difference between shoot length at the beginning and the end of the four-month experimental period was calculated for each species (∆SL = SL_f_ − SL_i_). Following harvest, the plant material was divided into aerial parts and roots using a cutter and then used for fresh biomass determination (SFW and RFW: shoot fresh weight and root fresh weight). Half of the aerial part was air-dried at room temperature and used for essential oil extraction. The other half of the aerial part was kept at −20 °C for enzymatic and biochemical investigation. The roots were thoroughly rinsed with running tap water and then distilled water until free of associated soil particles, and then kept in a plastic bag at −20 °C until the root architecture and morphology were analyzed.

### 4.4. Root Morphological Traits

The WinRHIZO™ software (2019a, Regent Instruments Inc., Quebec, QC, Canada) was used to quantify root morphological features, such as total root length (RL), average root diameter (RD), root surface area (RSA), and root volume (RV). Root fineness (RF) was also calculated by dividing the root length by root volume. Clean roots were dispersed to a depth of 1.5–2 cm in a Plexiglas tray filled with water and then scanned using an Epson Expression LA2400 scanner (Reagent Instruments Inc, Quebec, QC, Canada) at a resolution of 300 dpi. The collected images were processed with WinRHIZO™ software to determine root characteristics quantitatively. After the measurements, root fresh weight (RFW) and then the root dry weight (RDW) were determined after 72 h of drying at 80 °C.

### 4.5. Determination of Photosynthetic Pigments Content

For each replicate, 50 mg of fresh leaf biomass was homogenized in 2.5 mL 95% ethanol and left overnight at 4 °C to let the ethanol extract the maximum content of photosynthetic pigments present in the leaves extract. The final homogenate was vortexed for 30 s and centrifuged at 7000× *g* and 4 °C for 5 min. The analysis was performed in five replicates. The optic densities of all the samples were measured on the supernatant using a UV/visible Fluostar Omega multi-mode microplate reader (BMG Labtech, Ortenberg, Germany) at 649 nm, 665 nm, and 470 nm. Then, the chlorophyll a, b, and carotenoids contents were determined using the following methods described by [99,100]:

Chlorophyll *a* content (chl*a*) mg L^−1^ = 13.36 × (A665) − 5.19 × (A649) Chlorophyll *b* content (chl*b*) mg L^−1^ = 24.43 × (A649) − 8.12 × (A665) Carotenoids content (mg L^−1^) = [1000 × (A470) − (2.13 × chl*a*) − (97.64 × chl*b*)]/209

### 4.6. Total Soluble Sugars and Total Soluble Proteins

Shoot fresh samples (100 mg) were mixed with 1 mL distilled water in a homogenizer (Benchmark Scientific D2400 BeadBlaster™ 24, Sayreville, NJ, USA), extracted in an ultrasonic bath (Elmasonic S100, Elma Schmidbauer GmbH, Singen, Germany) for 30 min, and then filtered.

The method of Umbreit et al. [101] was used to determine total soluble sugars. A total of 100 µL of diluted extract (1/5) was combined with 0.9 mL of anthrone reagent for each replicate (0.2 g anthrone, 8 mL of ethanol absolute, 30 mL of distilled water, and 100 mL of sulfuric acid). The mixture was then heated at 95 °C in a water bath for 7 min. Using a UV/visible microplate reader (Fluostar Omega, BMG Labtech, Ortenberg, Germany), the intensity of the generated blue–green color was assessed at 620 nm. A standard curve was prepared using 0.01% glucose (*w/v*).

The measurement of soluble proteins was performed following the Bradford technique [102]. The measurement is conducted by adding 50 µL of reagent (Bio-Rad Laboratories, Richmond, CA, USA) to 200 µL of diluted extract (1/10). After 10 min, the optical density measurement on a UV/visible microplate reader (Fluostar Omega, BMG Labtech, Ortenberg, Germany) at 595 nm is obtained against a protein-free control. The soluble proteins concentration was determined using a standard curve of bovine serum albumin (BSA) as a reference protein. The analyses were performed in five replicates.

### 4.7. Determination of Antioxidant Enzymes Activities

For enzymatic extraction, 300 mg fresh shoot samples were homogenized in an ice bath with 3 mL of cold mixture containing 50 mM potassium phosphate buffer (pH 7.8), 2% (*w/v*) polyvinylpyrrolidone (PVP), and 0.2 mM ethylenediaminetetraacetic acid (EDTA). The homogenate was centrifuged for 20 min at 12,000 rpm at 4 °C (centrifuge: 521-1894 VWR Mega Star 600R, Leuven, Belgium). The supernatant was utilized to conduct APX (ascorbate peroxidase) and GuPx (guaiacol peroxidase) activity tests.

Ascorbate peroxidase activity was determined based on Nakano and Asada [103] and Boominathan and Doran [104] with slight modifications. The reaction solution was prepared using a mixture of 50 mM potassium phosphate buffer (pH 7) containing 0.2 mM EDTA and 0.5 mM ascorbic acid. An aliquot of 12.5 µL protein extract was mixed with 225 µL of reaction solution. Then, 12.5 µL of 2 mM H_2_O_2_ was added to initiate the reaction. On a UV/visible microplate reader (Fluostar Omega, BMG Labtech, Ortenberg, Germany) in kinetic mode, the absorbance reduction at 290 nm was recorded, and the enzyme activity was estimated using e = 2.6 mM^−1^ cm^−1^. One unit of ascorbate peroxidase is defined as the oxidation of 1 µmol min^−1^ of ascorbic acid at 25 °C.

Guaiacol peroxidase (GuPx) activity was measured using the method developed by Plewa et al. [105]. The reaction mixture (final volume 250 µL) contains 50 mM potassium phosphate buffer (pH 7), 0.3% (*v/v*) H_2_O_2,_ 1% (*v/v*) guaiacol, and 8 µL of the enzyme extract. The increase in absorbance was measured at 470 nm in a kinetic mode using a UV/visible microplate reader (Fluostar Omega, BMG Labtech, Ortenberg, Germany), and the enzyme activity was calculated using a molar extinction coefficient for tetraguaiacol of 25.5 mM^−1^ cm^−1^. The quantity of enzyme that generated 1 mmol min^−1^ of tetraguaiacol was defined as one unit of guaiacol peroxidase activity. The analyses were performed in five replicates.

### 4.8. Determination of Proline, Total Polyphenols, and Flavonoids Content

Shoot samples (50 mg) were mixed in 2.5 mL of 70% ethanol using a homogenizer (Benchmark Scientific D2400 BeadBlaster™ 24, Sayreville, NJ, USA). The extract was filtrated, and the filtrate was evaporated to obtain the extraction yield. The extract was used for proline determination, total phenolic and flavonoid contents, and total antioxidant capacity. The analyses were performed in five replicates.

Proline content was determined according to the method of Carillo and Gibon [106]. For each sample, 500 µL of the ethanolic extract was added to 1 mL of the reaction mixture containing 1% (*w/v*) ninhydrin, 60% (*v/v*) acetic acid, and 20% (*v/v*) ethanol. The mixture was heated at 95 °C on a heat block for 20 min. After centrifugation at 10,000× *g* for 1 min, the absorbance was measured at 520 nm (using a Fluostar Omega multi-mode microplate reader, BMG Labtech, Ortenberg, Germany). The proline content was calculated through the calibration curve of L-proline and the result was expressed as µg mg^−1^ of protein.

Content of total polyphenols was determined by the Folin–Ciocalteu method [107]. To 25 µL of the extract, 125 µL of fresh diluted Folin–Ciocalteu reagent (10%) was added and left to stand for 5 min prior to the addition of 100 µL of Na_2_CO_3_ solution (7.5% *w/v*). The tubes were incubated in the dark for 30 min, and the absorbance was read at 750 nm (using a Fluostar Omega multi-mode microplate reader, BMG Labtech, Ortenberg, Germany). A standard curve for total polyphenols content was prepared using gallic acid (GA) (0–100), and the results were expressed as the mg GA equivalent (eq) g^−1^ of dry weight.

The method of Chang et al. [108] was used to determine the flavonoids content. Briefly, 125 µL of extract was mixed with 62.5 µL of acetate potassium (120 mM) and 62.5 µL of aluminum chloride (1.2%). The mixture was allowed to react for 30 min, and the absorbance was taken at 415 nm (using a Fluostar Omega multi-mode microplate reader, BMG Labtech, Ortenberg, Germany). Flavonoids content was expressed as mg of quercetin (QE) eq g^−1^ of extract by dry weight through the calibration curve with quercetin (0–25).

### 4.9. Determination of Malondialdehyde Content

The concentration of malondialdehyde (MDA) generated by the thiobarbituric acid reaction was used to determine the lipid peroxidation as reported by Heath and Packer [109]. Fresh shoot (100 mg) was mixed in 1 mL of TCA (1%) using a homogenizer (Benchmark Scientific D2400 BeadBlaster™ 24, Sayreville, NJ, USA). After centrifuging the homogenized material at 10,000 rpm for 5 min, supernatant (0.5 mL) was mixed with 2.5 mL of 0.5% TBA. The mixture was heated at 95 C for 30 min, centrifuged at 5000 rpm for 5 min, and then the absorbance was measured at 532 and 600 nm. The blank was made up of 1% TBA and 20% TCA. The MDA concentration (nmol g^−1^ FW) was determined using an extinction coefficient e = 155 mM^−1^ cm^−1^. The analysis was performed in five replicates.

### 4.10. Essential Oil Extraction

The air-dried aerial parts of the four aromatic plants collected from the control soil and mine residue were subjected to hydrodistillation in a Clevenger-type apparatus and then extracted with 500 mL of distillated water for 8 h. The essential oils were collected, dried over anhydrous sodium sulfate, and filtered, then stored in sealed glass vials at 4 °C until determination of their chemical components.

### 4.11. Gas Chromatography and Mass Spectrophotometric (GC-MS) Analysis of Essential Oils

A Shimadzu Nexis GC-2030 instrument (Shimadzu Scientific Instruments, Inc., Columbia, MD, USA) coupled with a TQ8040 NX mass spectrometer (Shimadzu Scientific Instruments, Inc., Columbia, MD, USA) with a Restek RTX-5MS column (30 m length × 0.25 mm internal diameter, film thickness 0.25 µm) was used for the essential oil analyses of the four plants. The program temperature was initially set at 50 °C for 2 min before increasing to 300 °C at a rate of 5.5 °C/min and stabilizing for 3 min at 300 °C. Helium was used as a carrier gas with a flow rate of 1.5 mL/min, and the injector temperature was 250 °C (Injector HT2800T, HTA srl, Brescia, Italy). A volume of 1 µL of the diluted sample (1:10 *v/v* with *n*-hexane) was injected in a split mode (split ratio 50:1). The MS interface temperature was set at 280 °C, the ion source was set at 200 °C, and the scan of the mass range was from 50–500 *m*/*z*. The chemical compounds of essential oils were identified based on their retention indices (RI) calculated by interpolation to the C5–C24 (*n*-alkanes), and their mass spectra were compared to the compounds reported in the databases of NIST, namely the Wiley version libraries (National Institute of Standards and Technology) and Adams [110]. The peak area was used to estimate the percentage content of compounds.

### 4.12. Statistical Analysis

The statistical analysis was performed using IBM SPSS Statistics for Windows, version 20 (IBM Corp., Armonk, NY, USA). The normality of variables was assessed using Shapiro–Wilk’s normality test (*p* ≤ 0.05) and verified using skewness and kurtosis values. An independent *t*-test was used to compare the effect of growing soil on the aromatic plant analyses with a 95% interval of confidence. Additionally, the assumption of homogeneity of variances was evaluated and satisfied via Levene’s *F* test.

## 5. Conclusions

To summarize the results of this study, the main hypothesis was supported. This means that mining residue alone as growth substrate did not promote good plant growth. *R. officinalis* L. was the plant best able to cope with possible nutritional stress induced by MR, followed by *L. dentata* L. This result suggests that *R. officinalis* L. and *L. dentata* L. could be used for further testing in direct revegetation on phosphate mining residue. However, for better plant growth and development, phosphate mining residue characteristics needs to be improved. Our findings highlight the potential benefits of implementing soil improvement techniques to enhance soil fertility and structure, thereby creating a more conducive environment for plant growth and ecosystem restoration.

Recognizing the constraints imposed by the limited number of biological replicates, this study serves as an important first step in exploring the use of phosphate-mine residue as a growth medium for aromatic and medicinal plants. The results, while insightful, are preliminary, and future studies should incorporate larger sample sizes and extended field trials to confirm the findings in more realistic conditions. Moreover, long-term field experiments should focus on the sustainability of these plants in phosphate-mine residue over multiple growing seasons and should evaluate their potential contributions to soil stabilization, biodiversity enhancement, and mine site rehabilitation.

## Figures and Tables

**Table 1:** Soil textures of the phosphate-mine residue and the control soil used in the experiment.

Soil/Material	Texture	Particle Size Distribution (%)				
		Coarse Sand	Fine Sand	Coarse Silt	Fine Silt	Clay
MR	Sandy clay loam	29.78	28.39	9.96	10.16	21.71
C	Loam	18.93	22.30	14.67	23.29	20.81

C: Agriculture soil; MR: mine residue.

**Table 2:** Main physicochemical characteristics of the studied phosphate-mine residue and the control soil (0–15 cm).

Parameters	MR	C
pH	8.92 ± 0.02	8.27 ± 0.02
EC (mS cm^−1^)	0.56 ± 0.02	0.15 ± 0.01
OM (%)	0.51 ± 0.06	2.01 ± 0.06
P Olsen (mg P_2_O_5_ kg^−1^)	18.92 ± 2.70	45.67 ± 2.08
TKN (%)	0.03 ± 0.01	0.14 ± 0.01
Exchangeable potassium (mg K kg^−1^)	26.15 ± 0.07	162.28 ± 0.42

C: Agriculture soil; MR: mine residue; EC: electrical conductivity; OM: organic matter; P Olsen: available phosphorous; TKN: total Kjeldahl nitrogen.

**Table 3:** Change in the shoot humid and dry weight and the shoot length. ∆SL = SLf − SLi.

Species	Soil	SFW (g)	SDW (g)	∆SL (cm)
*L. dentata* L.	C	12.62 ^a^	2.63	15.00 ^a^
	MR	4.86 ^b^	1.87	9.50 ^b^
*R. officinalis* L.	C	4.61	1.55	8.45
	MR	4.40	1.48	8.40
*O. majorana* L.	C	6.85 ^a^	2.68 ^a^	9.00
	MR	1.40 ^b^	0.52 ^b^	8.50
*S. officinalis* L.	C	8.55 ^a^	1.89	7.63 ^a^
	MR	5.171 ^b^	1.501	3.00 ^b^

Values with superscript letters within each species differ significantly according to Student’s *t*-test between C and MR (*p* < 0.05). C: Agriculture soil; MR: mine residue; SFW: shoot fresh weight; SDW: shoot dry weight; ∆SL: delta shoot length.

**Table 4:** Variation in the morphological traits of root system of the aromatic and medicinal plants grown on the phosphate-mine residue.

Species	Soil	RFW (g)	RDW (g)	RL (cm)	RSA (cm^2^)	RAD (mm)	RV (cm^3^)	RF (cm cm^−3^)
*L. dentata* L.	C	3.606	0.970	1903.582	280.587	0.478	3.323	572.721
	MR	3.321	1.009	595.386	119.004	0.635	1.907	321.449
*R. officinalis* L.	C	2.77	1.216	1174.388	164.116	0.444	1.868	689.17
	MR	2.85	1.304	395.0666	75.829	0.615	1.175	349.486
*O. majorana* L.	C	2.591	0.881	1132.676	172.465	0.47	2.091	534.767
	MR	1.794	0.495	488.611	74.09	0.481	0.920	600.904
*S. officinalis* L.	C	3.679	0.989	1132.616	168.631	0.47	2.036	614.504
	MR	1.694	0.751	681.586	105.222	0.492	1.3	534.877

C: Agriculture soil; MR: mine residue; RFW: root fresh weight; RDW: root dry weight; RL: root length; RSA: root surface area; RAD: root average diameter; RV: root volume; RF: root fineness.

**Table 5:** Components of plant photosynthetic pigments.

Plant	Soil	Chl*a* (mg g^−1^ FW)	Chl*b* (mg g^−1^ FW)	Car (mg g FW)	Chl*a* + Chl*b*	Chl*a*/*b*	(Chl*a* + Chl*b*)/Car
*L. dentata* L.	C	0.367	0.111	0.026	0.478	3.309	18.520
	MR	0.217	0.078	0.034	0.295	2.804	8.596
*R. officinalis* L	C	0.362	0.103	0.051	0.465	3.514	9.101
	MR	0.361	0.115	0.061	0.476	3.139	7.792
*O. majorana* L.	C	0.650	0.157	0.068	0.807	4.147	11.815
	MR	0.343	0.110	0.025	0.453	3.121	17.828
*S. officinalis* L.	C	0.640	0.168	0.076	0.808	3.806	10.627
	MR	0.430	0.131	0.046	0.562	3.274	12.148

C: Agriculture soil; MR: mine residue; Chl: chlorophyll; Car: carotenoids.

## Data Availability

All data generated or analyzed during this study are included in this published article. Further information is available from the corresponding authors upon reasonable request.
